# Glycosyltransferases in Cancer: Prognostic Biomarkers of Survival in Patient Cohorts and Impact on Malignancy in Experimental Models

**DOI:** 10.3390/cancers14092128

**Published:** 2022-04-24

**Authors:** Michela Pucci, Martina Duca, Nadia Malagolini, Fabio Dall’Olio

**Affiliations:** Department of Experimental, Diagnostic and Specialty Medicine (DIMES), General Pathology Building, University of Bologna, Via San Giacomo 14, 40126 Bologna, Italy; michela.pucci3@unibo.it (M.P.); martina.duca3@unibo.it (M.D.); nadia.malagolini@unibo.it (N.M.)

**Keywords:** glycosyltransferases, glycosylation, Kaplan–Meier survival curves, TCGA, transcriptomic analysis

## Abstract

**Simple Summary:**

Cancer-associated glycosylation changes are widely used as biomarkers and strongly impact malignancy. However, the clinical significance of the deranged expression of glycosyltransferases observed in specimens is not always consistent with their role in experimental systems. We analyzed the overall survival curves of patients expressing high or low mRNA levels of 114 glycosyltransferases from the 21 cohorts of The Cancer Genome Atlas (TCGA). We identified 17 glycosyltransferases associated with poor prognosis and 4 associated with good prognosis in a large number of cohorts. In addition, we identified several glycosyltransferases with a very high prognostic value in only one or a few cohorts. Comparisons with published experimental works reveal partial consistency with TCGA clinical data. These data pave the way for the use of glycosyltransferases as prognostic markers and potential therapeutic targets and place experimental studies in an appropriate clinical context.

**Abstract:**

Background: Glycosylation changes are a main feature of cancer. Some carbohydrate epitopes and expression levels of glycosyltransferases have been used or proposed as prognostic markers, while many experimental works have investigated the role of glycosyltransferases in malignancy. Using the transcriptomic data of the 21 TCGA cohorts, we correlated the expression level of 114 glycosyltransferases with the overall survival of patients. Methods: Using the Oncolnc website, we determined the Kaplan–Meier survival curves for the patients falling in the 15% upper or lower percentile of mRNA expression of each glycosyltransferase. Results: Seventeen glycosyltransferases involved in initial steps of N- or O-glycosylation and of glycolipid biosynthesis, in chain extension and sialylation were unequivocally associated with bad prognosis in a majority of cohorts. Four glycosyltransferases were associated with good prognosis. Other glycosyltransferases displayed an extremely high predictive value in only one or a few cohorts. The top were GALNT3, ALG6 and B3GNT7, which displayed a *p* < 1 × 10^−9^ in the low-grade glioma (LGG) cohort. Comparison with published experimental data points to ALG3, GALNT2, B4GALNT1, POFUT1, B4GALT5, B3GNT5 and ST3GAL2 as the most consistently malignancy-associated enzymes. Conclusions: We identified several cancer-associated glycosyltransferases as potential prognostic markers and therapeutic targets.

## 1. Introduction

Glycosylation is a widely occurring modification of proteins and lipids that plays a crucial role in the modulation of cellular and molecular interactions [1]. Glycosylation is profoundly altered in cancer [2,3] and a huge number of clinical and experimental studies support the role of specific carbohydrate structures in determining cancer malignancy. However, studies performed in different experimental systems do not always provide consistent and reliable conclusions about the role of sugar chains and their cognate glycosyltransferases in cancer. On the other hand, clinical studies are often performed on small cohorts, which do not allow us to reach reliable conclusions on the impact of the overexpression of a glycosyltransferase on patient survival. The cancer genome atlas (TCGA) contains transcriptomic and clinical data from hundreds of patients affected by 21 malignancies. In this study, we determined the association between the level of expression of 114 glycosyltransferases in the 21 TCGA cohorts with patients’ overall survival. We identified a few glycosyltransferases whose high expression was unambiguously associated with a better or poorer prognosis in different cohorts. In addition, we identified glycosyltransferase with a very high prognostic value in one or a few cohorts. The role of the glycosyltransferases emerging from TCGA data analysis was compared with data obtained from experimental studies through an extensive literature review.

## 2. Glycosyltransferase Genes Associated with Prognosis in TCGA Cohorts

We first determined the association with the prognosis of 114 glycosyltransferases in all 21 TCGA cohorts. Table S1 reports the p value for the association with overall survival of the 15% upper percentile vs. the 15% lower percentile of glycosyltransferase mRNA expression, as obtained from the Oncolnc website. A dark red code label or a dark blue code label was assigned to significant (*p* ≤ 0.05) associations with a bad (red) or a good (blue) prognosis. A light red or light blue code label was assigned to strong but not significant associations (0.1 ≥ *p* ≥ 0.05). The percentage of glycosyltransferases significantly associated with overall survival was strikingly different in the different cohorts (Table S1, penultimate row). Obviously, a low number of patients in a cohort would make it harder to reach statistical significance. However, this was not the reason for the discrepancy. In fact, in the BRCA cohort, which is the most numerous (1006 cases Table S1, third row), only 16 glycosyltransferases displayed an association with prognosis (14%) (Table S1), while in the LGG cohort, which contains about half of the BRCA patients (510 cases), 69 glycosyltransferases were associated with prognosis (60%) (Table S1). For each cohort, one or more enzymes showing the lowest p value were identified as “best predictors” of bad (red) or good (blue) prognosis. Notably, MGAT4A and B4GALNT1 were best predictors of bad prognosis in 3 (ESCA, UCEC and LUSC) and 2 (HNSC and KIRP) cohorts, respectively.

## 3. Glycosyltransferase Genes Playing a Consistent Association in a Large Number of Cohorts

Several glycosyltransferase genes presented a prevalent association with a bad prognosis in a large number of cohorts, while a few displayed a prevalent association with a good prognosis. The former are referred to hereafter as “Bad Prognosis-associated”, (BPA) genes, while the latter as “Good Prognosis-associated”, (GPA) glycosyltransferases. Inclusion in either category was based on the difference between the number of cohorts in which it was associated with a bad prognosis and the number of cohorts in which it was related with a good prognosis. When this “score” reached a value ≥ 5, the glycosyltransferase was referred to as BPA, while GPA was referred to a glycosyltransferase with a score value ≤ −5. For example, ALG3 was associated with a bad prognosis in 11 cohorts and with a good prognosis in 2. Consequently, its BPA score was 9. According to this analysis, we identified 17 BPA and 4 GPA enzymes (Table 1).

Glycosyltransferases can be grouped as: initiating glycosyltransferases elaborating core structures of N- and O-linked chains and glycolipids; extending glycosyltransferases elongating sugar chains, which can be in common among N- and O-linked chains and glycolipids; and capping glycosyltransferases terminating sugar chains [4]. Table 2 reports the role of the 17 BPA and of the 4 GPA glycosyltransferases from Table 1 in glycan biosynthesis, as well as their score.

## 4. Glycosyltransferases with Very High Prognostic Value (VHPV)

**Figure 1:**
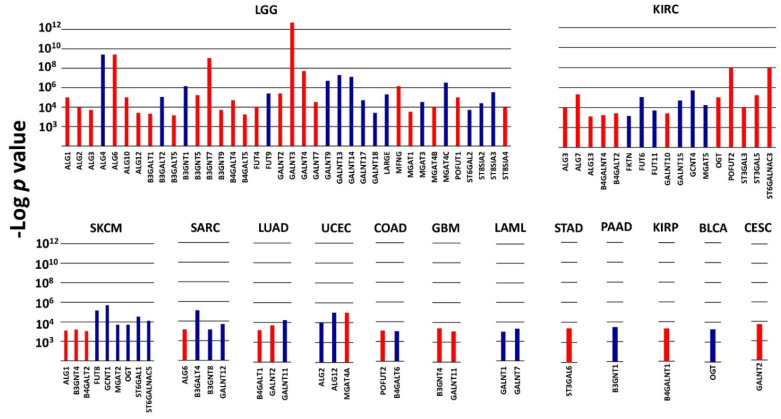
VHPV in TCGA cohorts. Histograms represent the −Log of the *p* value for the comparison between the overall survival curves of the 15% higher expressers of each glycosyltransferase gene and the 15% lower expressers. Color labels indicate the association with a bad (red) or good (blue) prognosis. *p* < 1 × 10^−3^ was arbitrarily set as the threshold limit for inclusion. Cohorts not present in the figure did not contain any VHPV enzymes.

**Figure 2:**
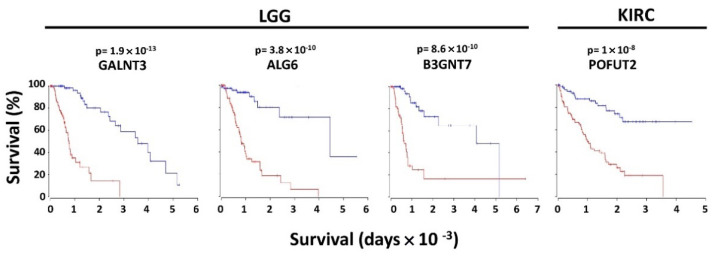
Kaplan–Meier of overall survival curves of the top four VHPV glycosyltransferases. Curves were determined by the Oncolnc website for the 15% higher (red) and 15% lower (blue) expressers of the four glycosyltransferases. LGG and KIRC refer to brain lower grade glioma and kidney clear cell carcinoma, respectively.

In the context of poor or good prognosis, several glycosyltransferases displayed a very high prognostic value (*p* ≤ 1 × 10^−3^) in a limited number of cohorts (Figure 1). These enzymes, which will be referred to as VHPV afterwards, were strikingly numerous in some cohorts. The cohort with the highest number of VHPV was LGG, followed by KIRC. Among the top 4 VHPV enzymes (*p* < 1 × 10^−8^), 3 were in LGG (GALNT3, ALG6 and B3GNT7), and 1 (POFUT2) was in KIRC (Figure 2). In LGG, a group of enzymes initiating N-glycosylation (ALG1 -2, -3, -6, -10, 12) or O-glycosylation (GALNT2, -3, -4, -7) displayed very strong association with poor prognosis. On the other hand, another group of GALNTs (9, 13, 14, 17, 18) showed a strong association with a good prognosis. Many VHPV glycosyltransferases displayed prognostic potential in only one cohort.

## 5. Role of Glycosyltransferases in Experimental Systems

The role of relevant glycosyltransferases, including BPA and GPA, in experimental systems was assessed through an extensive literature search.

### 5.1. Initiating Glycosyltransferases

#### 5.1.1. Glycosyltransferases Initiating N-Glycosylation

**Figure 3:**
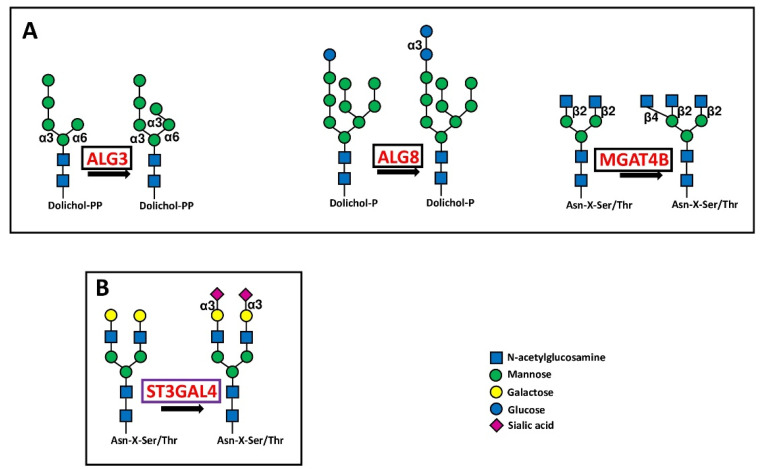
Biosynthetic steps in N-glycosylation. (**A**) shows the core glycosylation steps catalyzed by three BPA glycosyltransferases in initiation of N-glycosylation. (**B**) shows the reaction catalyzed by the chain capping sialyltransferase ST3GAL4 in N-glycan biosynthesis. Enzymes catalyzing core glycosylation are boxed in black, while that catalyzing chain capping is boxed in violet.

Glycosyltransferases ALG3, ALG8 and MGAT4B involved in the first steps of N-glycosylation behaved as BPA (Figure 3A).

Experimental data indicate that ALG3 contributes to malignancy of lung [5] and oral [6] cancer cell lines, in agreement with TCGA data reporting an association with a worse prognosis in LUAD and HNSC cohorts. However, the association with malignancy reported for esophageal [7] and cervical cancer [8] was not supported by TCGA data. ALG8 was reported to be associated with gastric [9] and colorectal [10] cancer. However, in the current study, we failed to observe any correlation with overall survival in these two malignancies. Very little or no information is available on the role of MGAT4B in experimental cancer systems.

#### 5.1.2. Glycosyltransferases Initiating O-Glycosylation

**Figure 4:**
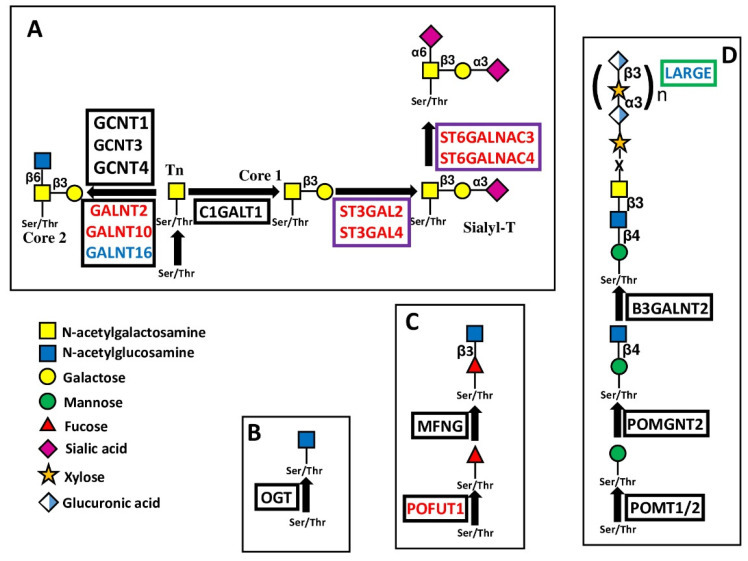
Biosynthesis and structure of O-linked chains. The sugar O-glycosidically linked to Ser/Thr can be GalNAc (**A**), as in Mucin-type O-glycosylation; GlcNAc (**B**), as in many cytosolic and nuclear proteins; fucose (**C**), as in NOTCH receptors; or mannose (**D**), as in dystroglycan. Enzymes catalyzing core glycosylation are boxed in black, those catalyzing chain extensions are boxed in green, while those catalyzing chain cappings are boxed in violet.

In the context of the 20 protein:O-GalNAc transferases mediating the addition of the first GalNAc of O-linked chains [11], GALNT2 and GALNT10 were identified as BPA. On the other hand, GALNT16 was GPA (Figure 4A). GALNT2, which is also the best predictor in CESC, provided a remarkable example of consistency between experimental data and prognosis. GALNT2 promoted malignancy through O-glycosylation of EGFR in oral cancer [12], glioma [13] and endometrial hyperplasia [14] cell lines, and by Notch signaling modulation [15] resulting in PD-L1 expression [16] in lung adenocarcinoma. Consistently, high GALNT2 expression was associated with poor overall survival in HNSC, LGG, UCEC and LUAD. On the other hand, increased malignancy related with high GALNT2 expression was also observed in hepatocellular carcinoma [17], while no relationship with overall survival was observed in the LIHC cohort. In gastric cancer cells, GALNT2 suppressed malignancy [18], but did not impact overall survival in STAD patients. Consistent with data of the OV cohort, in ovarian serous adenocarcinoma, high GALNT10 expression is related to an immunosuppressive microenvironment [19]. However, GALNT10 was causally associated with malignancy in cholangiocarcinoma [20] and hepatocellular carcinoma [21] but no relationship in the LIHC cohort was observed. Little or no information was available on GALNT16 in cancer.

Another type of O-glycosylation is O-GlcNAcylation (Figure 4B) [22]. The addition of a single O-GlcNAc residue to serine or threonine of cytosolic and nuclear proteins is mediated by a single enzyme, O-GlcNAc transferase (OGT). This enzyme is the best predictor of a good prognosis in BLCA. However, studies in bladder cancer cell lines have highlighted its tumor supporting activity [23,24].

A third type of O-glycosylation is O-fucosylation. POFUT1, which adds O-fucose to the NOTCH receptors (Figure 4C) [25], was found to be BPA. POFUT1, reported as a tumor-promoting glycosyltransferase in several studies, has also been proposed as a marker of colon cancer [26] and of high risk of tumor progression in adenomas [27]. Inhibition of POFUT1 decreased malignancy of CRC cell lines [28] by reducing stemness [29]. In a few cases, POFUT1 undergoes point mutation in CRC, resulting in enzyme hyperactivation and cancer progression [30]. POFUT2 is the best predictor of a poor prognosis in COAD. In hepatocarcinoma cells, POFUT1 promotes proliferation and invasion [31,32,33]. A high POFUT1 level correlates with glioblastoma [34] and lung [35], stomach [36], esophagus [37], breast [38], mouth [39] and bladder cancers [40]. However, only in the latter was an association with worse prognosis confirmed by TCGA data.

#### 5.1.3. Glycosyltransferases Initiating Gangliosides

**Figure 5:**
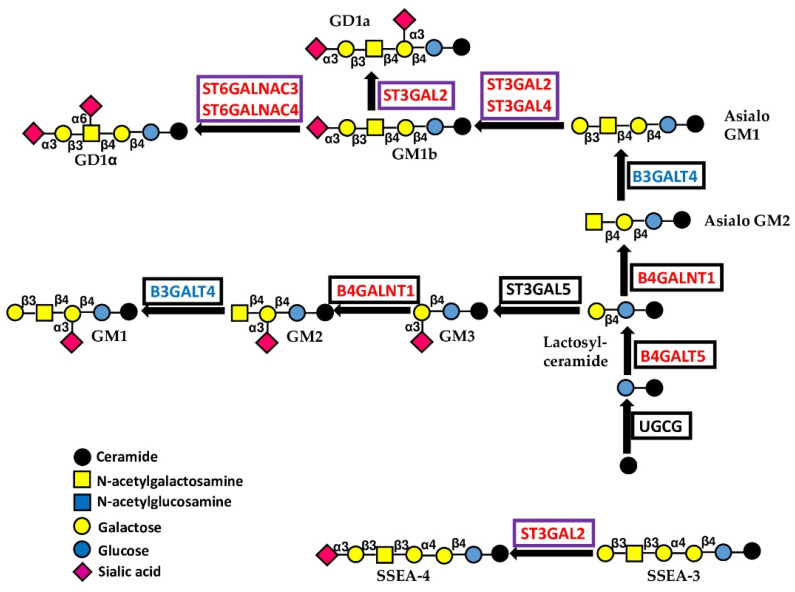
Biosynthesis and structure of gangliosides. The sugar linked to ceramide is glucose. Enzymes catalyzing core glycosylation are boxed in black, while those catalyzing chain capping are boxed in violet.

UGCG catalyzes the addition of glucose to ceramide (Figure 5). High expression of this enzyme increases malignancy in cervical cancer cells [41], consistent with the TCGA data of the CESC cohort (Table S1). B4GALT5 is the major enzyme involved in the biosynthesis of lactosyceramide, the root of all glycolipids [42,43], although it is also involved in other glycoconjugate formations. B4GALT5 increases the stemness and invasion of breast cancer cells [44] and multidrug resistance in leukemia cells [45]. No relationship with prognosis was evident in the BRCA cohort, while a tendency for a better prognosis was observed in the LAML cohort. B4GALNT1 catalyzes the synthesis of both GM2 and its asialo counterpart, asialo-GM2 (Figure 5). GM1, as well as GD2 and GD3, derive from GM2, while GD1a, GD1b and GD1α arise from asialo-GM2. Gangliosides GD2, GD3, GM2 and GD1a are greatly increased in breast cancer stem cells [46]. A causal correlation between high B4GALNT1 expression and malignancy has been noted in cell lines from lung, breast and kidney cancer, as well as in glioma and melanoma [46,47,48,49,50,51,52]. TCGA data are coherent with the B4GALNT1 role in kidney (KIRC) and lung (LUAD) cohorts. Phenotypically, the expression of B4GALNT1 has been associated with increased integrin signaling [52], reduced propensity to anoikis [49], stemness [46,50], augmented angiogenesis [51] and decreased immune surveillance [53]. B4GALNT1 is one of the most consistently and unambiguously glycosyltransferases associated with a bad prognosis (Table 1 and Table 2) in a large number of cohorts.

### 5.2. Extending Glycosyltransferases

Among the extending glycosyltransferases, we will consider the enzymes involved in polylactosamine biosynthesis and LARGE.

#### 5.2.1. Polylactosaminic Chains

**Figure 6:**
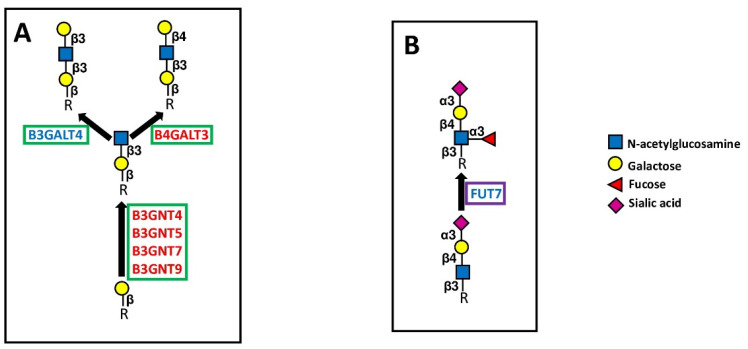
Extension and capping of chains. (**A**) shows the elongation of polylactosaminic chains. (**B**) shows the structure and biosynthesis of the sialyl Lewis x antigen, catalyzed by FUT7, as an example of chain capping. Elongating enzymes are boxed in green, and capping enzymes are boxed in violet.

Polylactosamines constitute repeated Gal-GlcNAc (lactosamine) units. The two sugars can be linked either by a β1,3 bond (type 1 chains) or by a β1,4 bond (type 2 chains) (Figure 6A). N-linked chains, as well as O-linked chains and glycolipids, can be elongated by polylactosaminic chains. The first step of their biosynthesis consists of the addition of a GlcNAc residue in β1,3 linkage to an underlying galactose (Figure 6A).

This reaction is mediated by different B3GNTs, specific to several types of sugar chains (e.g., type 2 chains for B3GNT4 and B3GNT7, glycolipids for B3GNT5, O-linked for B3GNT9). These four B3GNTs are BPA. B3GNT5 is a key enzyme for the biosynthesis of both type 1 and type 2 chains in glycolipids (Figure 6). Consistent with TCGA data, B3GNT5 enhances malignancy of glioma cells [54] and is stimulated by *Helicobacter pylori* infection in the stomach [55]. B3GNT7 promotes Lewis antigen expression [56] and suppresses malignancy in colon cancer cell lines [57], although in a large number of cohorts (but not in COAD), it is associated with worse prognosis. Within the B3GNTs group, B3GNT3 (which is neither a BPA nor a GPA) represents the subject of a larger number of studies. It mainly plays tumor-promoting activity in various types of tumors, including pancreatic [58,59], cervical [60], endometrial [61], and lung cancer [62,63]. In some instances, the expression of B3GNT3 inhibits the anti-cancer immune response, as in pancreatic [64], breast [65] and lung cancer [66]. In particular, in triple negative breast cancer, B3GNT3 promotes through EGFR the interaction between PD-1 and PD-L1, resulting in immune escape [65]. These insights are in good agreement with TCGA data, which report an association with poor prognosis in PAAD and LUAD cohorts. However, the tumor-suppressive role of B3GNT3 in pancreatic cancer [67] and neuroblastoma [68] has also been reported.

The second step in polylactosamine biosynthesis involves the addition of a galactose residue either through a β1,3 or a β1,4 linkage, generating type 1 or type 2 chains, respectively (Figure 6A). The enzyme B3GALT4, which both synthesizes type 1 chains and participates in ganglioside biosynthesis (Figure 5), is a GPA, although its association with poor survival in colon cancer has been reported [69]. On the other hand, the BPA B4GALT3 synthesizing type 2 chains behaves as a tumor-promoting gene in neuroblastoma [70,71], glioblastoma [72] and cervical carcinoma [73]. Consistently, B4GALT3 is a predictor of negative prognosis in the endometrial carcinoma (UCEC) cohort. However, B4GALT3 reduces malignancy in colon cancer [74].

#### 5.2.2. LARGE

α-Dystroglycan is a plasma membrane glycoprotein that indirectly links the cytoskeleton with the laminin of the extracellular matrix. The laminin- α-dystroglycan interaction is mediated by its peculiar O-mannosyl glycans [75,76]. The addition of mannose to the peptide is catalyzed by POMT1 and POMT2 (Figure 4D). The chain starting with the first O-linked mannose is elongated by other sugars and terminated by repeated disaccharide units comprised of xylose and glucuronic acid. The glycosyltransferase LARGE is responsible for the biosynthesis of these repeated disaccharide units. TCGA data show that in 6 cohorts, LARGE expression is associated with better prognosis. Although little data have been published on the relationship between LARGE expression and cancer, it has been described that O-mannosylation as a whole exerts tumor-suppressing activity in gastric cancer [77].

### 5.3. Capping Glycosyltransferases

#### 5.3.1. Sialyltransferases

The BPA sialyltransferases ST3GAL2, ST3GAL4, ST6GALNAC3 and ST6GALNAC4 are involved in the sialylation of both O-linked chains and glycolipids (Figure 4 and Figure 5), while ST3GAL4 sialylates also N-linked chains (Figure 3B). ST3GAL2 is differentially methylated in cancer [78] and is positively associated in oral cancer with advanced stages of the disease, lymph node involvement, and perineural invasion [79]. In addition to its involvement in sialylation of O-linked chains, ST3GAL2 is also a key player in ganglioside biosynthesis [80]. The ganglioside stage-specific embryonic antigen 4 (SSEA4), which is also a ST3GAL2 product, marks chemotherapy-resistant breast cancer cells with mesenchymal features [81]. Although not strictly associated with prognosis in BRCA and HNSC cohorts, the tumor-promoting activity of ST3GAL2 is supported by both experimental and clinical data. ST6GALNAC3 and ST6GALNAC4 are also involved in sialylation of both O-linked chains and glycolipids. ST6GALNAC3 was reduced in lung cancer tissues [82], while increased ST6GALNAC4 enhanced invasion of follicular thyroid carcinoma [83] and lung cancer [84]. Inconsistently, the latter is associated with a better prognosis in LUAD. ST3GAL6 is specific to type 2 chains and is the best predictor of poor survival in STAD. Experimental work has shown that its overexpression in gastric cancer cell lines protects against tyrosine kinase inhibitors [85].

#### 5.3.2. Fucosyltransferases

Fucosyltransferase FUT7 is one of the major α1,3 FUTs involved in the biosynthesis of the cancer-associated sialyl Lewis x antigen (Figure 6B). In this work, we observed that in the LAML cohort, high FUT7 was associated with worse prognosis, confirming a previous study [86]. Although in a variety of other malignancies, including lung [87,88], liver [89], bladder [90], thyroid [91], and breast [92] cancers FUT7 behaves as a tumor-promoting enzyme, in 7 of the TCGA cohorts, including LUAD, it is associated with better overall survival. In addition, FUT7 is the best predictor of good prognosis in BRCA.

## 6. Mechanistic Aspects of Glycosyltransferase Expression

Like other genes, glycosyltransferases are regulated at multiple levels, including the activity of specific transcription factors, promoter methylation, and the network of non-coding RNAs, such as micro RNA (miRNA), long non-coding RNAs (lnRNA) and circular RNAs (circRNA). On the other hand, glycosylated cell surface molecules, such as growth factor receptors and cell adhesion molecules, trigger multiple signaling pathways, resulting in modulation of cell behavior [93]. Table 3 reports the mechanisms regulating the expression of relevant glycosyltransferases (upstream regulators) and their downstream pathways.

From these data, it is evident that glycosyltransferases modulate different pathways in different cellular contexts. Sometimes, the activation of the same pathway induces opposite phenotypes in different tissues. For example, B4GALT3 activates β-integrin signaling in both neuroblastoma [71] and colon cancer [74], resulting in progression in the former and inhibition in the latter.

## 7. Discussion

The present work aims to combine the huge amount of clinical data from the public database TCGA with experimental studies on the glycosyltransferase role in cancer biology. Several key points emerged from the TCGA data analysis. First, some glycosyltransferases (BPA or GPA) are consistently associated with either poor or favorable prognosis in a large number of cohorts, while others (for example, ALG6 and GALNT12) displayed opposite associations in different cohorts. These findings support the notion that a few glycosyltransferases have a pleiotropic effect on several cell types and tissues, while the majority exert their effects in a tissue-specific manner. A paradigmatic example of this statement is provided by the B4GALNT2 gene, whose product synthesizes the carbohydrate antigen Sd^a^. A high level of B4GALNT2 expression is associated with longer overall survival in the COAD cohort and attenuation of malignant phenotype in colon cancer cell lines [105,106]. However, high B4GALNT2 expression correlated with a worse prognosis in the BRCA cohort [107] and increased malignancy in breast cancer cell lines [108]. Some BPA genes are involved in the early steps of N-glycosylation (ALG3, ALG8 and MGAT4B) and of mucin-type O-glycosylation (GALNT2 and GALNT10). Intriguingly, GALNT16, another member of the protein:O-GalNAc transferases, behaves as a GPA, indicating that subtle variations in the first step of O-glycosylation can lead to opposite effects on malignancy. The very strong association of POFUT1 with poor prognosis is probably due to its effect on the first step of NOTCH receptor glycosylation. The BPA group also includes enzymes involved in the biosynthesis of the core portion of glycolipids, such as B4GALT5 and B4GALNT1. B3GNT4, -5, -7 and -9, participating in initiation/extension of polylactosaminic chains, are also BPA, consistent with the recognized role of extended polylatosaminic chains in promoting malignancy. However, of the two galactosyltransferases synthesizing polylactosamines, the one producing type 2 chains (B4GALT3) is a BPA, while that producing type 1 chains (B3GALT4) is a GPA. The gene LARGE, responsible for the elongation of α-dystroglycan sugar chains, represents one of the stronger GPA, probably because of the role of its product in promoting cell adhesion. Among the capping enzymes, we identified 4 sialyltranferases acting mainly on glycolipids and/or O-linked chains behaving as BPA. This finding is not surprising, considering the well-established association of sialyltransferases with malignant phenotype [109,110]. By contrast, fucosyltransferases, another major class of capping enzymes, displayed an opposite behavior. This was unexpected, considering that several members of this group (FUT3-7) are responsible for the biosynthesis of well-known cancer-associated Lewis type antigens and their sialylated counterparts sialyl Lewis x and sialyl Lewis a [111]. FUT7 was found to be a GPA and a best predictor of good prognosis in BRCA, despite experimental studies showing its tumor-promoting activity. Several glycosyltransferases, including MGAT5 [112], FUT8 [113], ST6GAL1 [110], ST6GALNAC1 [114,115] and ST8SIA1 [116] have an established reputation as tumor-promoting enzymes. On the other hand, MGAT3 is probably the best-recognized tumor-restraining glycosyltransferase [117,118]. However, no one of these enzymes displays a relevant association with prognosis in different cohorts. Comparison of TCGA data with literature indicates a consistent malignancy-oriented behavior by some glycosyltransferases, including ALG3, GALNT2, B4GALNT1, POFUT1, B4GALT5, B3GNT5 and ST3GAL2. On the other hand, the profile of other glycosyltransferases emerging from TCGA data analysis appears to be inconsistent with that emerging from experimental studies. This group includes B3GALT5, B3GNT7, B3GALT4 and FUT7. The limited consistency between the experimental and clinical data could be explained by the fact that cell lines derived from a single or a few cancer cases might not be representative of the many patients of the whole cohort. Moreover, transcriptomic data are not necessarily representative of enzyme activity and cancer antigen expression levels. In fact, the biosynthesis of a given carbohydrate antigen is the final effect of many factors, including the translational efficiency of glycosyltransferase mRNA, the half-life of enzyme protein, the effect of postranslational modifications on enzmatic activity, the availability of donor and acceptor substrates, the competition with other glycosyltransferases and probably many others. In addition to the identification of glycosyltransferases playing a pleiotropic effect in many cohorts, we also pursued the identification of glycosyltransferases with a very high prognostic value (VHPV), in which the overall survival of the top 15% expressers was statistically different from that of the bottom 15% expressers with a *p* < 1 × 10^−3^. There were no VHPV glycosyltransferases in some cohorts, such as BRCA, while in others, such as LGG and KIRC, they were numerous. These discrepancies suggest that several tumors display intrinsically different sensitivity to glycosylation changes. We have shown that glycosyltransferases involved in the biosynthesis of different sugar chains are able to activate relatively few signal transduction pathways. EGFR/AKT appears to be one of the most frequently involved. Among the mechanisms regulating glycosyltransferase expression, the contribution of non-coding RNAs is increasingly recognized. The complex network of interactions between lncRNA, circRNA and miRNAs is essential to ensure the fine-tuning of glycosyltransferase expression. Considering the huge therapeutic importance of immune checkpoint inhibitors targeting the PD-1/PD-L1 interaction, it is worth mentioning that such interaction is modulated by glycosylation and that glycosylation inhibitors are able to revert the cancer-induced inhibition of the immune system [65,119,120,121,122,123,124].

## 8. Conclusions

In conclusion, the wide analysis of TCGA data allows the identification of glycosyltransferases whose over- or under-expression impacts patients’ overall survival more dramatically. Even if the studies on experimental systems remain crucial to understanding the molecular mechanisms linking glycosyltransferase expression and malignancy, information from databases appears to be the best way to identify glycosyltransferases as potential biomarkers, either alone or in combination [125,126,127,128]. Owing to their very strict association with survival in specific malignancies, VHPV glycosyltransferases are ideal candidates as prognostic biomarkers and targets of therapeutic approaches.

## Figures and Tables

**Table 1:** Prognostic value in TCGA cohorts of BPA and GPA glycosyltransferases.

	ALG3	ALG8	B3GALT4	B3GNT4	B3GNT5	B3GNT7	B3GNT9	B4GALNT1	B4GALT3	B4GALT5	FUT7	GALNT2	GALNT10	GALNT16	LARGE	MGAT4B	POFUT1	ST3GAL2	ST3GAL4	ST6GALNAC3	ST6GALNAC4
**BRCA**																					
**HNSC**																					
**ESCA**																					
**STAD**																					
**COAD**																					
**READ**																					
**LIHC**																					
**PAAD**																					
**KIRC**																					
**KIRP**																					
**BLCA**																					
**CESC**																					
**UCEC**																					
**OV**																					
**LUAD**																					
**LUSC**																					
**GBM**																					
**LGG**																					
**SKCM**																					
**SARC**																					
**LAML**																					

Association with overall survival of the 15% upper percentile vs. the 15% lower percentile of glycosyltransferase mRNA expression, as obtained from the Oncolnc website. The dark red code label or a dark blue code label indicates a significant (*p* ≤ 0.05) associations with a bad (red) or a good (blue) prognosis. A light red or blue code label indicates a strong tendency but not significant associations (0.1 ≥ *p* ≥ 0.05). BPA is marked in red, while GPA is marked in blue.

**Table 2:** Glycosyltransferases show an association with prognosis in a large number of cohorts.

Pathway	Enzyme	Activity	Product	Score
Core N-glycosylation	ALG3	α1,3-mannosyltransferase	Mannosylated precursor	9
	ALG8	α1,3-glucosyltransferase	Glucosylated precursor	**7**
	MGAT4B	β1,4 GlcNAc transferase B	β1,4-branched N-glycans	6
Core O-glycosylation (mucin type)	GALNT2	Protein:O-GalNAC transferase 2	Tn-antigen	8
	GALNT10	Protein:O-GalNAC transferase 10	Tn-antigen	5
	GALNT16	Protein:O-GalNAC transferase 16	Tn-antigen	−6
O-fucosylation	POFUT1	Protein O-fucosyltransferase 1	O-fucosylated NOTCH	**7**
Core of Glycolipids	B4GALT5	β1,4-Galactosyltransferase 5	Lactosylceramide	8
	B4GALNT1	β1,4-GalNAc transferase 1	Ganglioside GM2, asialo GM2	**8**
Chain extension	B3GNT4	β1,3-GlcNAc transferase 4	Type 2 polylactosaminic chains	5
	B3GNT5	β1,3-GlcNAc transferase 5	Lactotriaosylceramide	7
	B3GNT7	β1,3-GlcNAc transferase 7	Type 2 polylactosaminic chains	6
	B3GNT9	β1,3-GlcNAc transferase 9	Polylactosamines O-linked	**5**
	B4GALT3	β1,4-Galactosyltransferase 3	Type 2 lactosaminic chains	**7**
	B3GALT4	β1,3-Galactosyltransferase 4	Type 1 lactosaminic chains	−5
O-mannosylation	LARGE	Xylosyltransferase and β1,3-glucuronyltransferase	Elongated O-mannosyl glycans	**−6**
Capping	ST3GAL2	α2,3 to Gal sialyltransferase 2	Sialyl-T; Gangliosides GD1a, GM1b, GT1b	**6**
	ST3GAL4	α2,3 to Gal sialyltransferase 4	Sialyl-T; N-glycans; Gangliosides GD1a, GM1b	6
	ST6GALNAC3	α2,6 to GalNAc sialyltransferase 3	Di-sialyl T; Gangliosides GD1α, GM1b	6
	ST6GALNAC4	α2,6 to GalNAc sialyltransferase 4	Di-sialyl T; Ganglioside GD1α	5
	FUT7	α1,3/6 fucosyltransferase 7	Sialyl Lewis X	−6

BPA and GPA have positive or negative score values, respectively. Scores marked in bold refer to those enzymes that were associated with bad or good prognosis in all the cohorts with predictive value.

**Table 3:** Mechanistic aspects of glycosyltransferase action and regulation.

Enzyme	Upstream Regulator(s)	Downstream Pathways	Cancer Tissue/Cell Line	Effect *	
ALG3		TGF-β receptor 2	Breast	Stemness, radioresistance	[94]
	Heat shock factor 2		Breast	Progression	[95]
	miR-98-5p		Non-small cell lung	Progression	[5]
B3GNT3		RhoA/RAC1	Endometrial	Progression	[61]
	miR-149-5p		Lung	Progression	[62]
		EGFR/PD-L1	Lung	Immune escape	[66]
		EGF/PD1-PD-L1	Breast	Immune escape	[65]
B3GNT5	lncRNA MIR44352HG/miR1365p		Liver	Progression	[96]
B3GNT7	Promoter methylation		Colorectal	Inhibition **	[57]
B4GALT3	lncRNA DANCR/miR-338-3p		Neuroblastoma	Progression	[70]
		β1-integrins	Neuroblastoma	Progression	[71]
	miR-27a	β1-integrins	Cervical	Progression	[73]
		β1-integrins	Colorectal	Inhibition	[74]
B4GALT5		Wnt/β-catenin	Breast	Stemness	[44]
	Circ_0009910/miR-491-5p	PI3K/AKT	Acute myeloid leukemia	Progression	[97]
B4GALNT1		JNK/c-Jun/Slug	Lung	Progression	[47]
		EGFR	breast	Stemness	[50]
		β1 integrins/FAK/SRC/ERK	Glioblastoma, lung, kidney	Progression	[52]
		GM2/GD2	Melanoma	Angiogenesis, progression	[51]
GALNT2		IGF-R1	Neuroblastoma	Inhibition	[98]
		Met	Gastric	Inhibition	[18]
		EGFR	Liver	Inhibition	[17]
		EGFR/AKT	Oral squamous	Progression	[12]
		EGFR/PI3K/AKT/mTOR	Glioma	Progression	[13]
		Notch/Hes1-PTEN-PI3K/AKT	Lung	Progression	[15]
GALNT10	DLGAP1-AS2/miR-505		Bile ducts	Progression	[20]
	HNF4/miR-122		Liver	Progression	[21]
	Histone methylation/ZBTB2		Ovary	Stemness	[99]
FUT7		EGFR/AKT/mTOR	Lung	Progression	[88]
	Promoter methylation		Bladder	Progression	[90]
		EGFR	Thyroid	Progression	[91]
POFUT1		Notch	Liver	Progression	[31,32]
			Colorectal		[28,29]
			Glioblastoma		[34]
ST3GAL4		Met, RON	Gastric	Progression	[85,100,101]
	Promoter methylation/GATA2		Ovary, Breast	Progression	[102,103]
	miR-370		Colorectal	Adhesion	[104]
ST6GALNAC4	miR-429		Thyroid	Progression	[83]

* The indicated effect is positively related to the expression of the indicated glycosyltransferase. ** Inhibition indicates attenuation of the neoplastic phenotype.

## Supplementary Materials

The following supporting information can be downloaded at: https://www.mdpi.com/article/10.3390/cancers14092128/s1, Table S1: Prognostic value of glycosyltransferase gene expression in TCGA cohorts.

## Abbreviations

BPA	bad prognosis-associated
circRNA	circular RNA
GPA	good prognosis-associated
lncRNA	long non-coding RNA
PD-1	programmed death 1
PD-L1	programmed death ligand 1
VHPV	very high prognostic value
BLCA	bladder urothelial carcinoma
BRCA	breast invasive carcinoma
CESC	cervical squamous cell carcinoma and endocervical adenocarcinoma
COAD	colon adenocarcinoma
ESCA	esophageal carcinoma
GBM	glioblastoma multiforme
HNSC	head and neck squamous cell carcinoma
KIRC	kidney renal clear cell carcinoma
KIRP	kidney renal papillary carcinoma
LAML	acute myeloid leukemia
LGG	brain lower grade glioma
LIHC	liver hepatocellular carcinoma
LUAD	lung adenocarcinoma
LUSC	lung squamous cell carcinoma
OV	ovary serous cystadenocarcinoma
PAAD	pancreatic adenocarcinoma
READ	rectum adenocarcinoma
SARC	sarcoma
SKCM	skin cutaneous melanoma
STAD	stomach adenocarcinoma
TCGA	the cancer genome atlas
UCEC	uterine corpus endometrial carcinoma
